# Blast Transformation of Chronic Myeloid Leukemia Driven by Acquisition of t(8;21)(q22;q22)/*RUNX1::RUNX1T1*: Selecting Optimal Treatment Based on Clinical and Molecular Findings

**DOI:** 10.3390/biomedicines12102339

**Published:** 2024-10-15

**Authors:** Adolfo Fernández-Sánchez, Alberto Hernández-Sánchez, Cristina De Ramón, María-Carmen Chillón, María Belén Vidriales, Mónica Baile-González, Cristina-Teresa Fuentes-Morales, Magdalena Sierra-Pacho, Lucía López-Corral, Fermín Sánchez-Guijo

**Affiliations:** Hematology Department, IBSAL-University Hospital of Salamanca, Department of Medicine and Cancer Research Center (CIC), University of Salamanca, 37007 Salamanca, Spain

**Keywords:** chronic myeloid leukemia, blast phase, core binding factor rearrangement, T315I mutation

## Abstract

The advent of tyrosine kinase inhibitors (TKIs) has changed the natural history of chronic myeloid leukemia (CML), and the transformation from the chronic phase to the blast phase (BP) is currently an uncommon situation. However, it is one of the major remaining challenges in the management of this disease, as it is associated with dismal outcomes. We report the case of a 63-year-old woman with a history of CML with poor response to imatinib who progressed to myeloid BP-CML, driven by the acquisition of t(8;21)(q22;q22)/*RUNX1::RUNX1T1*. The patient received intensive chemotherapy and dasatinib, followed by allogeneic hematopoietic stem cell transplantation (allo-HSCT). However, she suffered an early relapse after allo-HSCT with the acquisition of the T315I mutation in *ABL1*. Ponatinib and azacitidine were started as salvage treatment, allowing for the achievement of complete remission with deep molecular response after five cycles. Advances in the knowledge of disease biology and clonal evolution are crucial for optimal treatment selection, which ultimately translates into better patient outcomes.

## 1. Introduction

Since the advent of tyrosine kinase inhibitors (TKIs), chronic myeloid leukemia (CML) has become a paradigm of successful targeted treatment for hematologic malignancies [1]. The inhibition of *BCR::ABL1* tyrosine kinase activity has transformed the natural history of a disease with a very poor prognosis for patients who could not undergo early allogeneic hematopoietic stem cell transplantation (allo-HSCT) into a generally controllable entity, allowing most chronic-phase patients to achieve a normal life expectancy [2]. In fact, a subgroup of patients can be eligible for treatment discontinuation after reaching and maintaining a deep molecular response [3]. However, progression to blast phase (BP), an aggressive form of acute leukemia with an unfavorable prognosis, may occur in up to 5% of patients [4]. Despite the major advances in chronic-phase (CP) CML, the outcome is still dismal for a minority of patients that develop BP [5]. The treatment of BP with TKIs and conventional chemotherapy often leads to short-term remissions, with most patients experiencing a disease relapse if allo-HSCT is not performed [6,7]. Due to the rarity of this condition and the lack of clinical trials, most of the available recommendations are based on retrospective analyses and expert consensus, so further studies are required [8,9]. The acquisition of cytogenetic abnormalities (ACAs) is frequently considered to be the driver event of BP transformation, although the acquisition of t(8;21) is rare in this context, and to our knowledge, only three cases have been described to date. For this reason, we report an exceptional case of a patient with a history of CML who progressed to myeloid BP driven by the acquisition of t(8;21)(q22;q22)/*RUNX1::RUNX1T1* within 6 months from the initial diagnosis, which highlights the importance of understanding the biology and pathogenesis of hematological diseases to offer the best available treatment.

## 2. Detailed Case Description

We report the case of a 63-year-old woman, without antecedents of interest, who underwent a routine blood test where hyperleukocytosis of 288.9 × 10^9^/L was observed, accompanied with mild anemia (hemoglobin 11.5 g/dL) and platelets in the normal range (179 × 10^9^/L). The peripheral blood smear showed marked left deviation with 25% segmented neutrophils, 20% band neutrophils, 6% metamyelocytes, 23% myelocytes, 6% promyelocytes, 12% medium-large immature cells, 15% monocytes and 17% lymphocytes. The patient was asymptomatic, and the physical examination was unremarkable, without lymph node enlargements, splenomegaly or masses.

### 2.1. High-Risk Chronic Myeloid Leukemia

The bone marrow was hypercellular at the expense of the granulocytic and megakaryocytic series, with the presence of 5% immature cells. The fluorescence in situ hybridization (FISH) study demonstrated the presence of t(9;22)(q34;q11)/*BCR::ABL1* (Philadelphia-positive) translocation, which was also confirmed in the peripheral blood sample. Quantitative real-time polymerase chain reaction (RT-qPCR) with Taqman probes confirmed the presence of the *BCR::ABL1* major (p210) fusion gene with a *BCR::ABL1/ABL1* ratio of 194.22% according to the International Scale (IS). Therefore, a diagnosis of chronic myeloid leukemia (CML) in chronic phase was established, according to the criteria of the European LeukemiaNet (ELN) [10]. Next-generation sequencing (NGS) analysis performed with a custom Pan-Myeloid Panel (Sophia Genetics, Switzerland) [11] revealed a mutation in *DNMT3A* (c.C2206G, p.R736G, VAF 48%) and two mutations in *TET2* (c.1340delG, p.R447fs, VAF 47% and c.1495delC, p.P499fs, VAF 45%).

At diagnosis, the patient had the following prognostic scores: Sokal 2.87 (high risk) [12], Hasford 1588.8 (high risk) [13], Eutos 0.1 (low risk) and ELTS 3.17 (high risk) [14,15].

Treatment was started with imatinib at a dose of 400 mg per day, with good initial tolerance and no evidence of tumor lysis syndrome (TLS).

After three months of treatment with imatinib, a *BCR::ABL1/ABL1*^IS^ ratio of 81.56% was observed, and treatment failure was confirmed one month later with a ratio of 96.94% [16]. Second-line treatment was started with dasatinib at 100 mg/day, as the Sanger sequencing of the *BCR::ABL1* kinase domain was negative at this point. Two weeks after starting dasatinib, the patient developed progressive cytopenias, which were initially attributed to drug toxicity, so the dose was reduced to 50 mg/day and subsequently discontinued.

### 2.2. Transformation from Chronic to Blast Phase

**Figure 1:**
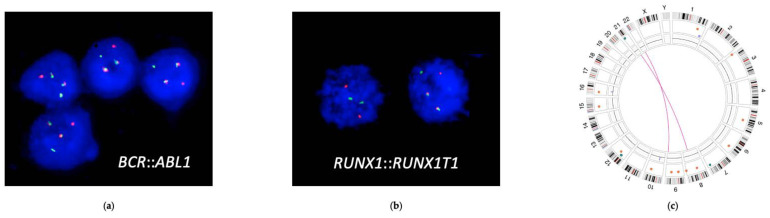
Cytogenetic studies of the patient at myeloid BP-CML, showing the acquisition of t(8;21)(q22;q22)/*RUNX1::RUNX1T1* as the driver event of BP. (**a**): *BCR::ABL1* fusion probe (Dual Color, Dual Fusion, Vysis LSI) in the FISH study, showing two fusions in most of the cells, representative of the presence of t(9;22)(q34.1;q11.2)/*BCR::ABL1* (**b**): *RUNX1::RUNX1T1* fusion probe (Dual Color, Dual Fusion, Vysis LSI) in the FISH study, showing two fusions in approximately half of the cells, representative of the presence of t(8;21)(q22;q22)/*RUNX1::RUNX1T1* (**c**): A Circos plot of optical genome mapping (Bionano) showing concomitant t(8;21)(q22;q22)/*RUNX1::RUNX1T1* and t(9;22)(q34.1;q11.2)/*BCR::ABL1* in the patient sample.

One week after discontinuation, the patient was admitted to the emergency department for fever and abdominal pain. The blood test showed the worsening of the cytopenias with a hemoglobin level of 8.8 g/dL, leukocytes 8.6 × 10^9^/L (monocytes 5.3 × 10^9^/L), platelets 41 × 10^9^/L and an LDH level of 522 U/L (normal laboratory range: 120–246 U/L). A peripheral blood smear revealed the presence of 21% myeloid blasts, so the patient was admitted to the hematology ward to characterize the BP transformation. The bone marrow study showed the presence of 41% of myeloid immature cells, and by using multiparameter flow cytometry (MFC), two immature cell populations were observed: a more immature CD34+ population (19%) with early monocyte lineage differentiation and a more mature CD34−/+ population (22%) with early basophil differentiation. Moreover, the monocytic cells showed the reversal of the CD14/CD300e pattern and the aberrant expression of CD56 in 45% of them. The FISH study showed the translocation t(9;22)(q34;q11)/*BCR::ABL1* in 90% of the cells and, strikingly, the acquisition of translocation t(8;21)(q22;q22)/*RUNX1::RUNX1T1* in 45% of them. Quantitative RT-PCR with Taqman probes showed the presence of the *BCR::ABL1* major (p210) fusion gene with an increased ratio in both bone marrow (ratio of 182.81%) and peripheral blood (ratio of 221.75%), and it also showed the presence of the *RUNX1::RUNX1T1* fusion gene with 562 copies of the normalized altered gene compared to 10,000 copies of the control gene *ABL*. The absence of mutations in the *ABL1* gene was confirmed again. The concomitant presence of t(9;22)(q34;q11) and t(8;21)(q22;q22) was also confirmed by optical genome mapping (*Bionano*) (Figure 1). NGS analysis with the myeloid panel was repeated, confirming the persistence of mutations in *DNMT3A* and *TET2* observed at the diagnosis of CML, with similar VAF, without additional abnormalities.

Intensive chemotherapy treatment was initiated with the FLAG-IDA scheme (fludarabine, cytarabine, idarubicin and granulocyte colony-stimulating factor), together with dasatinib 140 mg/day. As complications developed after intensive chemotherapy, the patient presented TLS, acute pulmonary edema and two episodes of febrile neutropenia, which were successfully resolved.

On day +21 after starting FLAG-IDA + dasatinib, the first re-evaluation of the disease was performed with a cytomorphological study showing bone marrow aplasia without blasts and positive minimal residual disease (MRD) by MFC (3.45% blasts) and by RT-qPCR with a *BCR::ABL1/ABL1*^IS^ ratio of 250.72% and 467 normalized copies of the *RUNX1::RUNX1T1* transcript.

A new bone marrow re-evaluation was performed on day +30 with the persistence of positive MRD by MFC with 1.5% immature cells and MRD not evaluable by RT-qPCR due to an insufficient sample. Of note, in this bone marrow study, the presence of 0.6% of pathological plasma cells was observed with 92% of them having an aberrant immunophenotype and 1q gain by FISH after plasma cell enrichment performed on CD138+ cells. Subsequently, a complete study of monoclonal gammopathy was performed. A monoclonal IgA kappa peak was detected by immunofixation with kappa light chains of 331 mg/L and an altered kappa/lambda ratio of 50, as well as Bence Jones proteinuria in urine (120 mg in 24 h). Positron Emission Tomography with Computed Tomography (PET/TC) was negative, and therefore, a diagnosis of concomitant monoclonal gammopathy of undetermined significance (MGUS) was established.

Due to the availability of an HLA-matched related donor, a peripheral blood allo-HSCT with reduced-intensity conditioning with fludarabine, busulfan and thiotepa was performed. Graft-versus-host disease (GVHD) prophylaxis consisted of tacrolimus plus sirolimus plus mycophenolate. As early complications after allogeneic transplantation, the patient developed digestive mucositis grade 3 and an episode of febrile neutropenia, which were successfully resolved [17].

The first re-evaluation after allo-HSCT was performed on day +21, and the patient achieved complete response with incomplete hematologic recovery and negative MRD by MFC, although they were still positive by RT-qPCR with a *BCR::ABL1/ABL1*^IS^ ratio of 0.4883% and 1.38 normalized copies of the *RUNX1::RUNX1T1* transcript. Donor chimerism was complete in both bone marrow and CD3+ peripheral blood.

### 2.3. Early Relapse after Allogeneic Stem Cell Transplantation

On re-evaluation on day +56 after allo-HSCT, MRD was higher, with 0.8% immature cells by MFC, and a significant increase in the *BCR::ABL1/ABL1*^IS^ ratio of 30.5% and in *RUNX1::RUNX1T1* transcripts (198 normalized copies) was observed; donor chimerism remained complete. Treatment with dasatinib was restarted at a dose of 70 mg per day, awaiting the results of the third *ABL1* mutational study.

**Figure 2:**
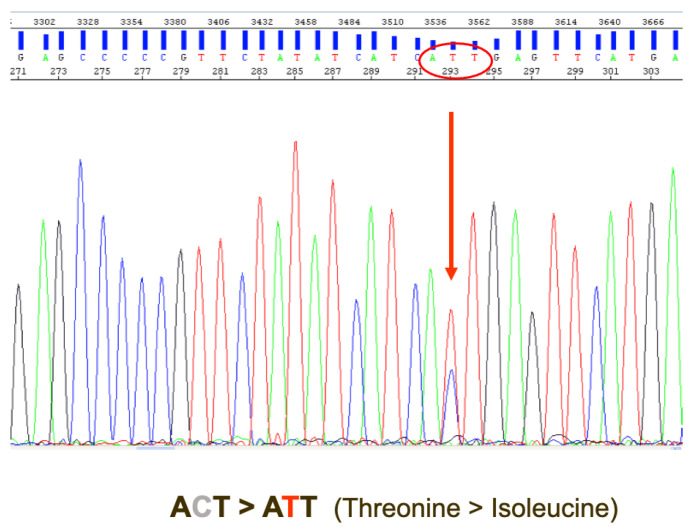
Polymerase chain reaction and Sanger sequencing analysis of T315I mutation in *BCR::ABL1* fusion gene (c.944C > T, p.Thr315Ile).

An additional bone marrow study was performed on day +68, where there was a persistent increase in the detection of blast cells by MFC (1.68%), as well as in the *BCR::ABL1/ABL1*^IS^ ratio (86.40%) and *RUNX1::RUNX1T1* transcripts (334 normalized copies). The mutational study of *ABL1* revealed the presence of the T315I kinase domain mutation that confers resistance to imatinib, nilotinib, dasatinib and bosutinib (Figure 2) [8,18]. Therefore, the TKI was changed from dasatinib to ponatinib 45 mg per day, immunosuppression was tapered and the patient was started on azacitidine treatment (75 mg/m^2^ × 7 days each 28 days).

On re-evaluation after the first cycle with azacitidine, a loss of chimerism with 12.5% of recipient hematopoiesis in bone marrow and 6.8% in CD3+ peripheral blood, an increase in immature cells in MFC reaching 4.9% and an increase in *BCR::ABL1/ABL1*^IS^ ratio (145.70%) and *RUNX1::RUNX1T1* transcripts (814 normalized copies) were observed.

**Figure 3:**
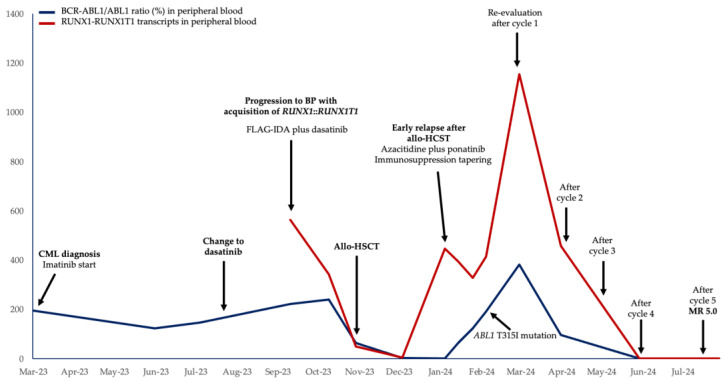
A summary of the clinical case report. The temporal evolution of the *BCR::ABL1/ABL1* ratio according to the International Scale (represented in blue) and the number of *RUNX1::RUNX1T1* transcripts (represented in red) in peripheral blood samples are shown, together with the main events that the patient presented during the disease evolution. CML: chronic myeloid leukemia; BP: blast phase; FLAG-IDA: fludarabine, cytarabine, idarubicin and granulocyte colony-stimulating factor; allo-HCST: allogenic hematopoietic stem cell transplantation; MR: molecular response.

However, in the successive cycles of azacitidine together with ponatinib treatment, a response was achieved, recovering complete chimerism in bone marrow and peripheral blood after two cycles, negative MRD by MFC after three cycles and negative MRD by RT-qPCR in the *BCR::ABL1/ABL1*^IS^ ratio and *RUNX1::RUNX1T1* transcripts both in bone marrow and in peripheral blood after four cycles. No pathological plasma cells were detected again in bone marrow re-evaluations. The evolution of the *BCR::ABL1/ABL1*^IS^ ratio and *RUNX1::RUNX1T1* transcripts during the disease course is summarized in Figure 3, showing the 16-month evolution from the initial diagnosis. Genetic alterations that the patient presented during the disease course are summarized in Table 1.

After immunosuppression withdrawal, the patient developed moderate chronic GVHD with liver, skin and oral involvement which completely resolved with topical and systemic steroid treatment [19].

More than one year after developing myeloid BP-CML with concomitant t(9;22)(q34;q11) and t(8;21)(q22;q22), the patient remains in complete response with negative MRD by MFC and RT-qPCR, reaching a grade 5.0 molecular response [16].

## 3. Discussion

We reported an uncommon case of a patient with CML that progressed to BP with the acquisition of core binding factor (CBF) rearrangement, who presented a concomitant MGUS and suffered an early relapse after allo-HSCT due to the acquisition of the *ABL1* T315I mutation. The patient was successfully rescued with optimized targeted therapy.

The blast phase of chronic myeloid leukemia still represents an unmet clinical need as it is associated with a dismal prognosis [20]. Due to the infrequent presentation and the heterogeneity in the biology of the condition, prospective trials and consensus treatment recommendations are scarce [9]. Several clinical, cytogenetic and molecular features have been identified as prognostic factors in BP-CML. These include older age (>58 years), thrombocytopenia (<100 × 10^9^/L), anemia (hemoglobin < 10 g/dL), high LDH (above normal range), prior TKI therapy, progression from CP, myeloid immunophenotype and the development of ACAs [21,22]. Most of these adverse prognostic factors were present in our patient, highlighting the clinical challenge of this scenario.

The therapy choice for BP-CML depends on several factors: de novo presentation or progression after CP-CML, immunophenotype, prior TKIs that the patient has been exposed to, the presence of *ABL1* mutations and whether the patient is fit for intensive chemotherapy and allo-HSCT [20]. Monotherapy with TKIs can induce complete cytogenetic remissions in 10–45% of patients in BP with minimal toxicity, but these remissions are typically short-lived [23]. Conventional chemotherapy regimens, such as FLAG-IDA, can induce remissions in 30–40% of patients who have progressed to BP, but again, most patients relapse within six months, and survival is poor [24]. A logical approach proposed in several studies is to combine both strategies. This is supported by the synergism between TKIs and chemotherapy, which have been proven to be successful in the treatment of other hematological malignancies, including B-cell acute lymphoid leukemia with *BCR::ABL1* fusion [25,26].

After reviewing the literature, eight articles were found presenting the results of different case series combining a TKI with different types of intensive chemotherapy for myeloid BP-CML. The number of patients included in each study varies between 7 and 36 patients. The results are heterogeneous, with hematological complete responses between 19 and 81%, cytogenetic complete responses between 10 and 50% and median overall survival between 5 and 27 months. Response was consolidated with allo-HSCT in 26% to 70% of patients, depending on the publication [27,28,29,30,31,32,33,34]. Patients who received allo-HSCT had the best responses and the best overall survival. Therefore, although further studies are needed, the combination of a TKI with intensive chemotherapy followed by allo-HSCT seems to be the best approach in this challenging situation [35]. Some clinical trials are currently underway, exploring the combination of TKIs with different drugs (e.g., decitabine or venetoclax) in non-intensive treatment approaches. These drug combinations are safe, although further studies are needed [32,36,37].

Regarding the association of CBF rearrangement in BP-CML, it is important to stress that ACAs can be observed in 3–7% of patients with CML at the time of diagnosis, but they are more frequent at disease progression to BP (60–80%) [38,39]. Therefore, the acquisition of cytogenetic abnormalities is sometimes considered to be the driving event in BP transformation. Adverse ACAs include trisomy 8; an extra Philadelphia chromosome; isochromosome 17q; trisomy 17, 19 or 21; 3q26.2 or 11q23 rearrangements; −7/7q abnormalities and complex karyotypes. Patients with CML and adverse ACAs have a poor outcome with a 5-year overall survival (OS) of 50% [38]. However, the prognostic impact of other ACAs has not been established yet.

CBF leukemia is a subtype of acute myeloid leukemia (AML) characterized by the presence of either reciprocal translocation between the long arms of chromosomes 8 and 21, t(8;21)(q22;q22)/*RUNX1::RUNX1T1*, pericentric inversion of chromosome 16, inv(16)(p13q22), or its variant, t(16;16)(p13q22)/*CBFB::MYH11* [40]. CBF AML is generally considered to have a favorable prognosis, and most patients are able to achieve a long-lasting complete remission of the disease without allo-HSCT [41]. However, the prognostic impact of CBF rearrangements in CML is not well established. To our knowledge, the largest cohort reported to date was in a retrospective analysis of 11 patients, where CBF rearrangement was proposed as a high-risk ACA, as it was associated with a median overall survival of 6 months. Remarkably, 91% of those patients presented with inv(16)(p13q22), whereas t(8;21)(q22;q22) was found in only one patient in CP-CML [42]. Therefore, the acquisition of *RUNX1::RUNXT1* at BP-CML is extremely infrequent, and only a few isolated cases have been reported to date [43,44,45].

In view of the above, it is reasonable to consider CBF rearrangement as the driver of the transformation to BP in our patient. A retrospective analysis was performed in the CP sample of our patient that ruled out the initial presence of the CBF rearrangement. In addition, the NGS study did not reveal the presence of new mutations, as the variants observed at CP-CML (*TET2* and *DNMT3A*) remained unchanged at BP-CML with similar VAF. Approximately 35% of patients with CP-CML have mutations at diagnosis, and the most frequent is the *ASXL1* gene mutation (10%). Variants in *DNMT3A* are described in 2% of patients, and *TET2* mutations are also reported in 2%, increasing to 5% in patients in BP [46,47].

Regarding the association of MGUS with concomitant CML in this case, it is important to mention that the association of plasma cell dyscrasias with some myeloid hematological malignancies is well established, including Philadelphia-negative chronic myeloproliferative neoplasms [48]. However, its association with CML is unclear. The cases reported in the literature are usually patients with CP-CML on TKI maintenance therapy who develop different types of plasma cell dyscrasias, including multiple myeloma, during the course of their disease [49,50]. To our knowledge, there is only one retrospective analysis of 100 patients studying the association of CML and paraproteinemia that observed a higher prevalence of paraproteinemia (6% vs. 3.2%) in patients with CML, although further studies with larger sample sizes are needed to confirm this possible association [51].

Allo-HSCT currently represents the only potentially curative approach for many patients with high-risk hematological malignancies. The availability and outcome of allo-HSCT have progressively improved over the last few decades due to advances in donor selection, transplantation procedures and supportive care. However, the relapse of AML after allo-HSCT still represents a clinical challenge, with few therapeutic options. Long-term survival in transplanted patients with AML relapse has been reported to be less than 10%, highlighting the need for new treatments [52].

On the one hand, some studies have revealed that relapsed myeloid neoplasm after allo-HSCT could express a higher level of gene methylation. Hypomethylating agents can promote the expression of tumor antigens and induce CD8^+^ T cells to recognize silenced tumor-associated antigens, ultimately exerting antitumor effects in myeloid neoplasms. Therefore, azacitidine may play a role in the treatment of those patients, together with the rapid decrease in immunosuppression in search of an early graft-versus-leukemia effect [53].

On the other hand, it is mandatory to look for mutations in the *BCR::ABL1* kinase domain in patients who relapse under TKI treatment. The most important mechanism of resistance against TKIs is the selection of leukemic clones driven by *BCR::ABL1* point mutations, such as E255K, Y253F/H (P-loop), H396R (activation loop) or T315I (gatekeeper). The “gatekeeper” mutation T315I confers resistance to most of the available TKIs [8,18]. Ponatinib was the first multi-target kinase inhibitor to overcome T315I mutation, showing efficacy when this mutation is present, with promising results in BP-CML [54]. In relation to our case, the study of *BCR::ABL1* mutations was performed at three time points: the absence of response to imatinib, progression to BP and early relapse after allo-HSCT. However, the T315I mutation was observed only at relapse following allo-HSCT. Treatment with FLAG-IDA and dasatinib could have selected a resistant clone, which emerged right after allo-HSCT and was responsible for the early relapse driven by a clone that still carried t(8;21)(q22;q22)/*RUNX1::RUNX1T1*.

The identification of the T315I mutation was crucial for starting salvage treatment with ponatinib and azacitidine, which managed to achieve complete disease remission and deep molecular response.

## 4. Conclusions

CML is a paradigm of successful targeted treatment disease and usually has a favorable long-term prognosis. Nevertheless, a small proportion of patients still progress to the blast phase, highlighting the importance of continuous monitoring. The best treatment for BP-CML to date is intensive chemotherapy together with TKIs followed by allogeneic transplantation. The acquisition of ACAs is common in patients with progression to BP, although the acquisition of t(8;21)(q22;q22)/*RUNX1::RUNX1T1* is exceedingly rare. Relapse after allogeneic transplantation is a challenging situation, in which it is essential to test again for *ABL1* mutations, as the identification of the T315I variant guided the optimal targeted treatment with ponatinib. Advances in the understanding of disease biology and the mechanisms of resistance to treatment can provide crucial information to achieve disease remission in the challenging scenario of myeloid BP-CML.

## Figures and Tables

**Table 1:** A summary of genetic alterations that the patient presented during the disease. CML: chronic myeloid leukemia; BP: blast phase; allo-HCST: allogenic hematopoietic stem cell transplantation; NGS: next-generation sequencing.

CML Diagnosis (March 2023)	CML Progression to Myeloid BP (September 2023)	Relapse after allo-HSCT (January 2024)
- t(9;22)(q34;q11)/*BCR::ABL1*	- t(9;22)(q34;q11)/*BCR::ABL1*	- t(9;22)(q34;q11)/*BCR::ABL1*
	- t(8;21)(q22;q22)/ *RUNX1::RUNX1T1*	- t(8;21)(q22;q22)/ *RUNX1::RUNX1T1*
		- *ABL1* T315I
- *DNMT3A* R736G (VAF 47.7%)	- *DNMT3A* R736G (VAF 44.3%)	NGS panel not performed
*- TET2* R447fs (VAF 47.2%) - *TET2* P499fs (VAF 45%)	*- TET2* R447fs (VAF 46.3%) - *TET2* P499fs (VAF 45%)	

## Data Availability

The data presented in this study are available on request from the corresponding author. The data are not publicly available due to patients’ privacy.
